# Supplementary material for: Comprehensive Analysis of the Prognostic Significance of the TRIM Family in the Context of TP53 Mutations in Cancers
Source: Cancers (Basel). 2023 Jul 26;15(15):3792. doi: 10.3390/cancers15153792 (PMC10417774; doi:10.3390/cancers15153792)
Supplement: Supplementary file 1 [file cancers-15-03792-s001.zip › cancers-2424200-supplementary.pdf]

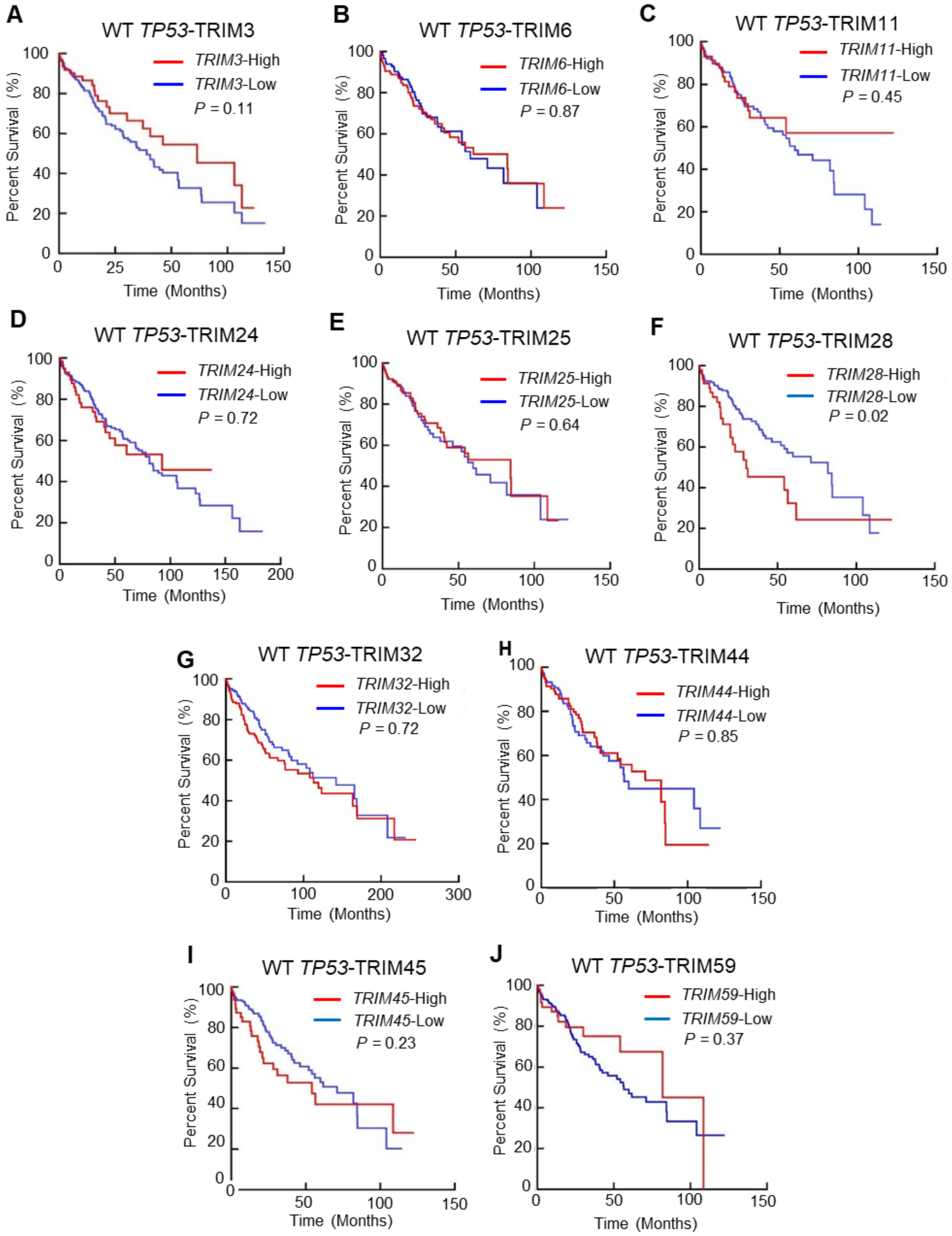

Figure S1: The prognostic values of the nine TRIM family members in the Wildtype (WT) TP53 LIHC. (A-J) Kaplan–Meier analysis estimates of overall survival based on the expression levels of TRIM3 (A), TRIM6 (B), TRIM11 (C), TRIM24 (D), TRIM25 (E), TRIM28 (F), TRIM32 (G), TRIM44 (H), TRIM45 (I), and TRIM59 (J) in TCGA-LIHC patients.

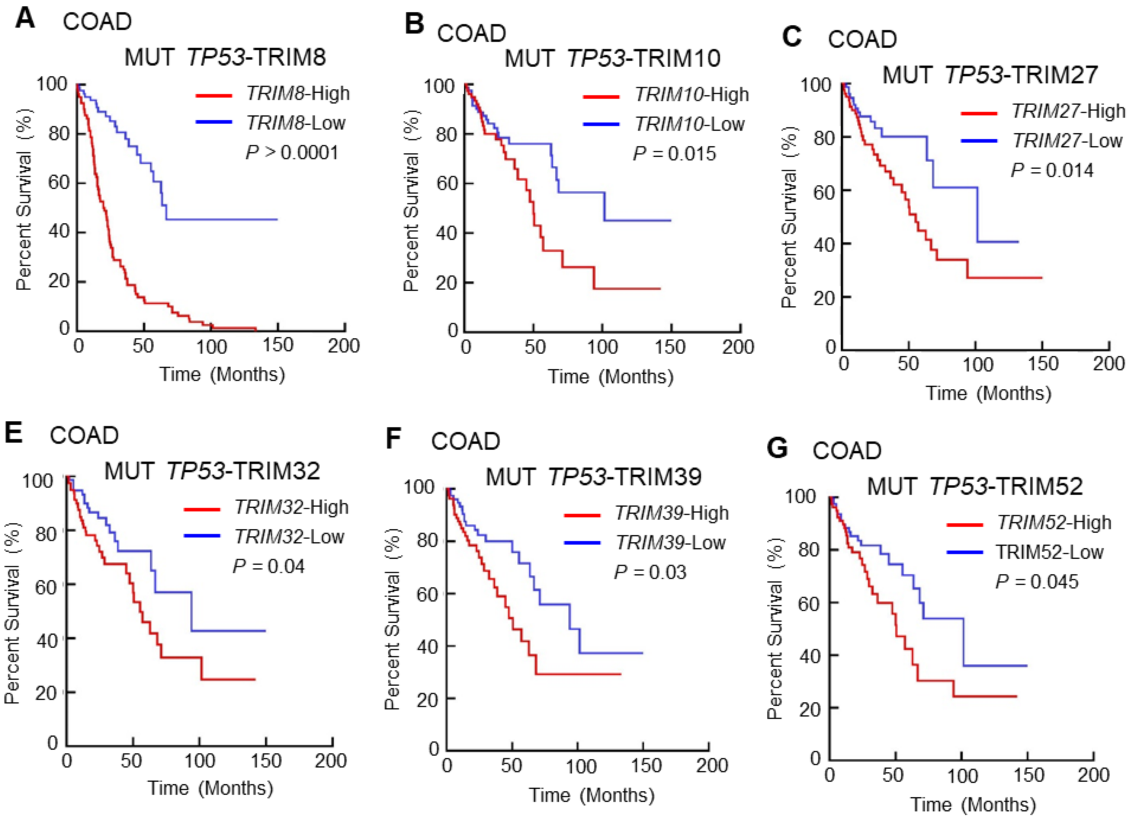

Figure S2: The prognostic values of the nine TRIM family members in the mutant (Mut) TP53 COAD. (A-F) Kaplan–Meier analysis estimates of overall survival based on the expression levels of TRIM8 (A), TRIM10 (B), TRIM27 (C), TRIM32 (D), TRIM39 (E), and TRIM52 (F) in TCGA-LIHC patients.

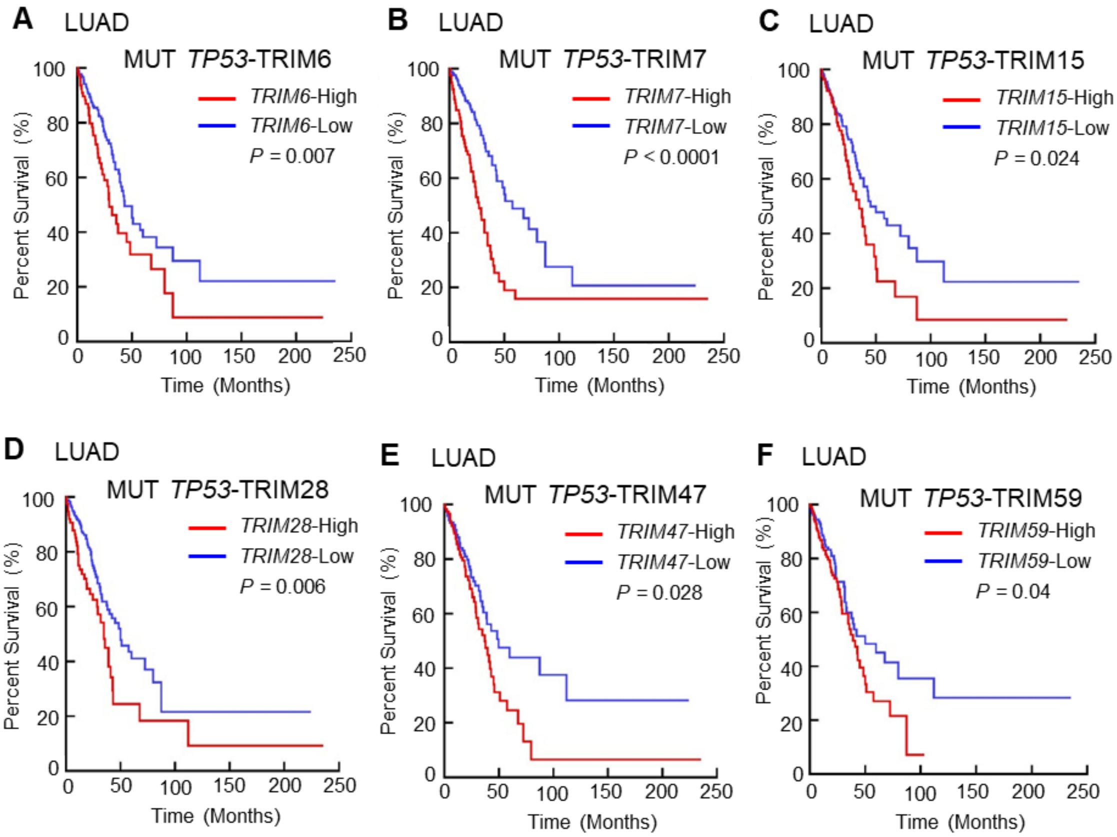

Figure S3: The prognostic values of the nine TRIM family members in the mutant (Mut) TP53 LUAD. (A-F) Kaplan–Meier analysis estimates of overall survival based on the expression levels of TRIM6 (A), TRIM7 (B), TRIM15 (C), TRIM28 (D), TRIM47 (E), and TRIM59 (F) in TCGA-LIHC patients.

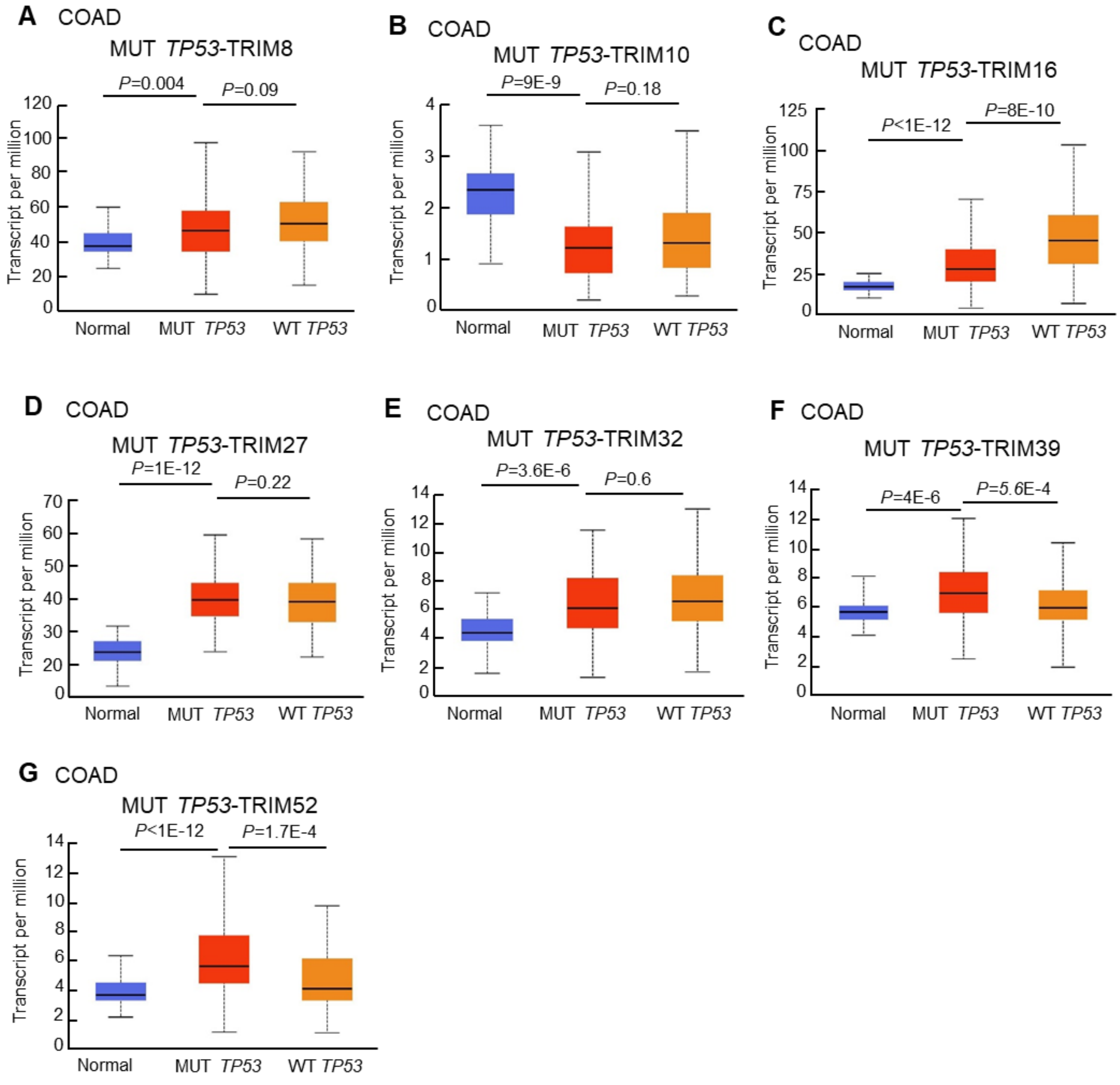

Figure S4: The association between mRNA levels of TRIM genes and TP53 mutation in COAD. The transcriptional levels of TRIM8 (A), TRIM10 (B), TRIM16 (C), TRIM27 (D), TRIM32(E), TRIM39 (F), and TRIM52(G) in normal liver tissues (Blue), and mutant TP53 (Red) and wild-type TP53 (Orange) liver cancer tissues (UALCAN).

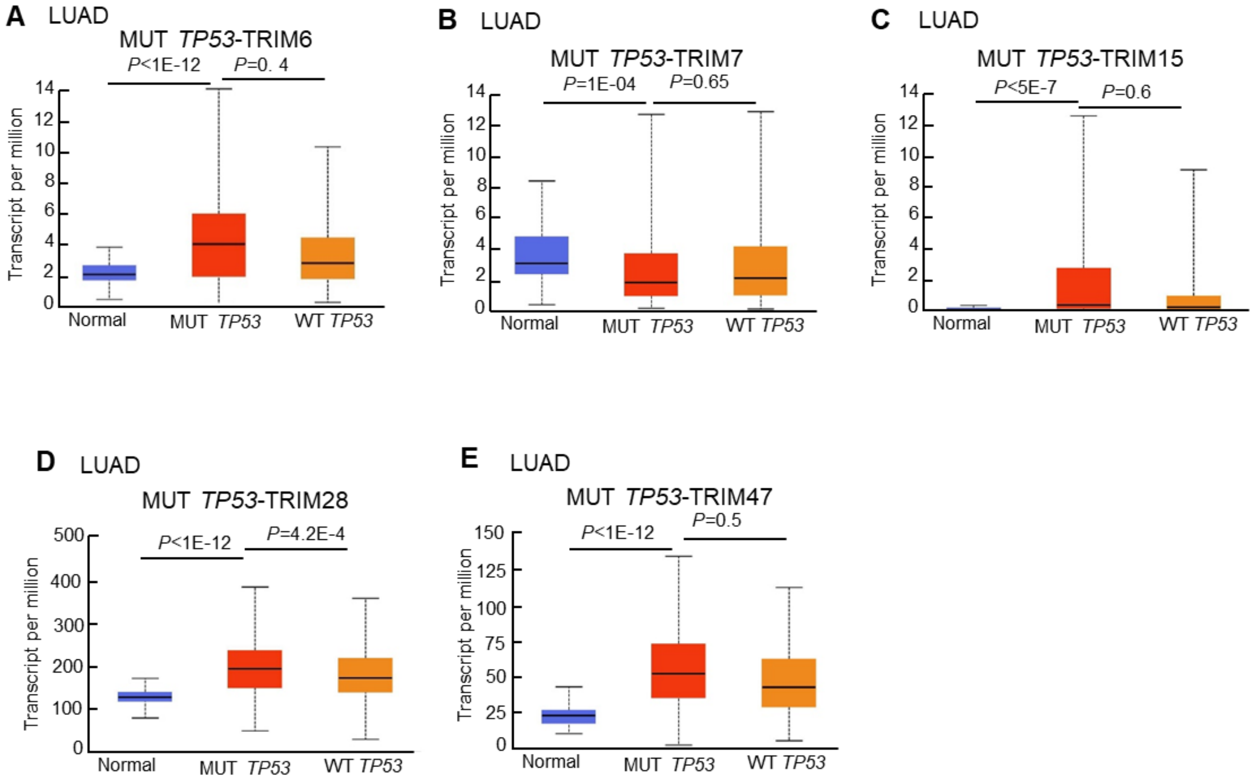

Figure S5: The association between mRNA levels of TRIM genes and TP53 mutation in LUAD. The transcriptional levels of TRIM6 (A), TRIM7 (B), TRIM15 (C), TRIM28 (D), and TRIM47 (E) in normal liver tissues (Blue), and mutant TP53 (Red) and wild-type TP53 (Orange) liver cancer tissues (UALCAN).

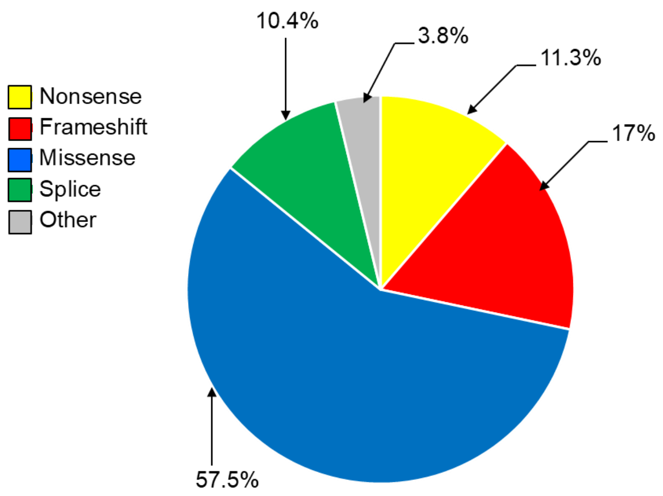

Figure S6: Pie chart showing the proportion of different types of somatic TP53 mutations.

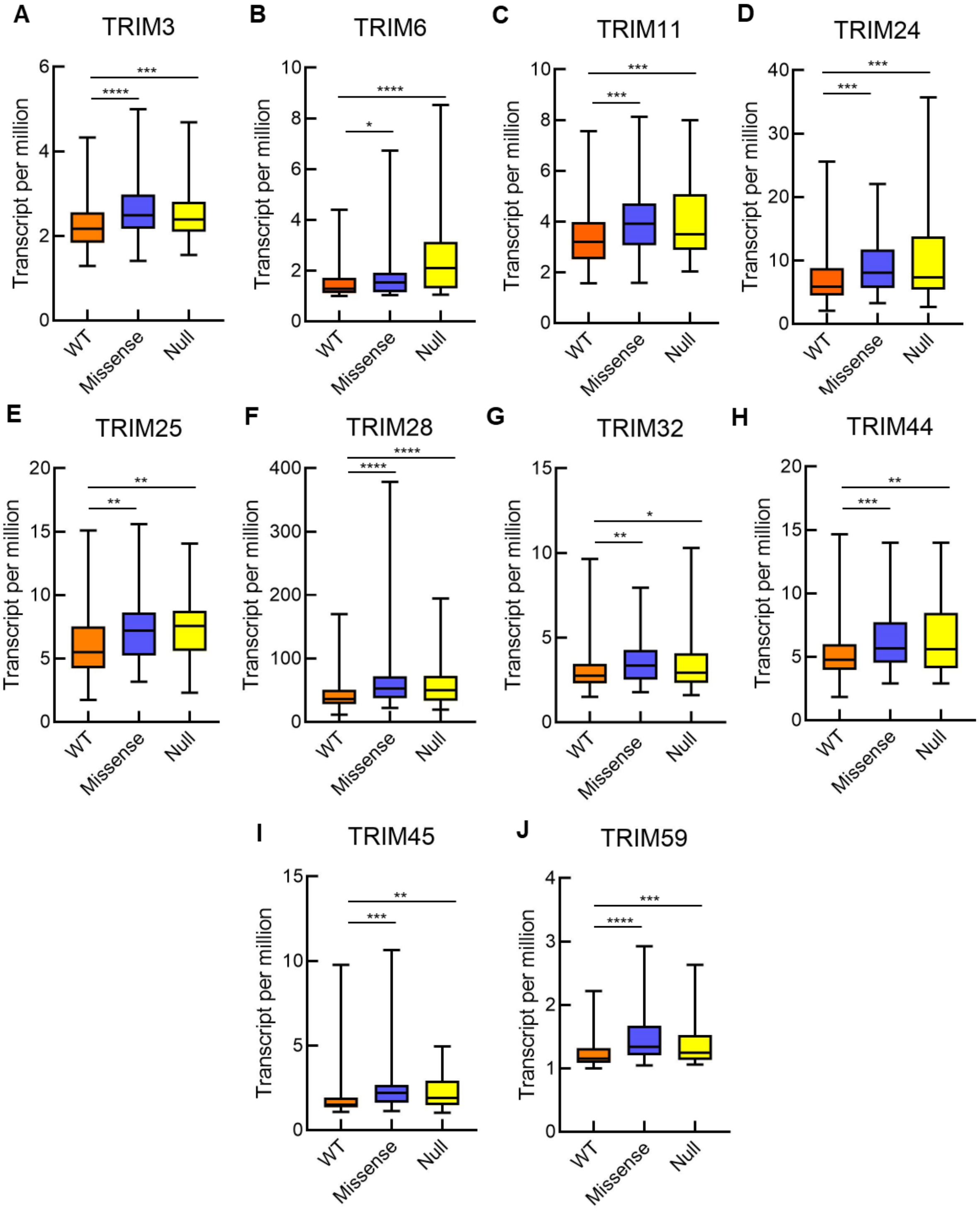

Figure S7: LIHC samples from TCGA were divided into WT p53, p53-null (frameshift and nonsense TP53 mutations), and p53 missense mutated tumors. The transcriptional levels of TRIM3 (A), TRIM6 (B), TRIM11 (C), TRIM24 (D), TRIM25 (E), TRIM28 (F), TRIM32 (G), TRIM44 (H), TRIM45 (I), and TRIM59 (J) in wildtype TP53 (Orange), p53 missense mutated (Blue), and p53 null (Yellow) LIHC tumors.

	TRIM3	TRIM6	TRIM11	TRIM24	TRIM25	TRIM28	TRIM32	TRIM44	TRIM45	TRIM59
E2F_TARGETS	-1.15	-1.2	0.67	1.61	0.95	1.14	1.27	-2.85	1.25	0.74
G2M_CHECKPOINT	-1.05	0.79	0.73	1.35	1.1	1.39	0.69	-1.2	1.15	1.12
MITOTIC_SPINDLE	0.84	0.85	1.25	0.85	-0.85	0.95	0.77	1.28	0.85	1.12

Figure S8. Heatmaps of normalized enrichment scores (NES) calculated by GSEA against the Hallmark cell cycle, G2/M checkpoint control, and E2F target gene sets of genes upregulated in wildtype TP53 tumors with increased expression of TRIM family members.

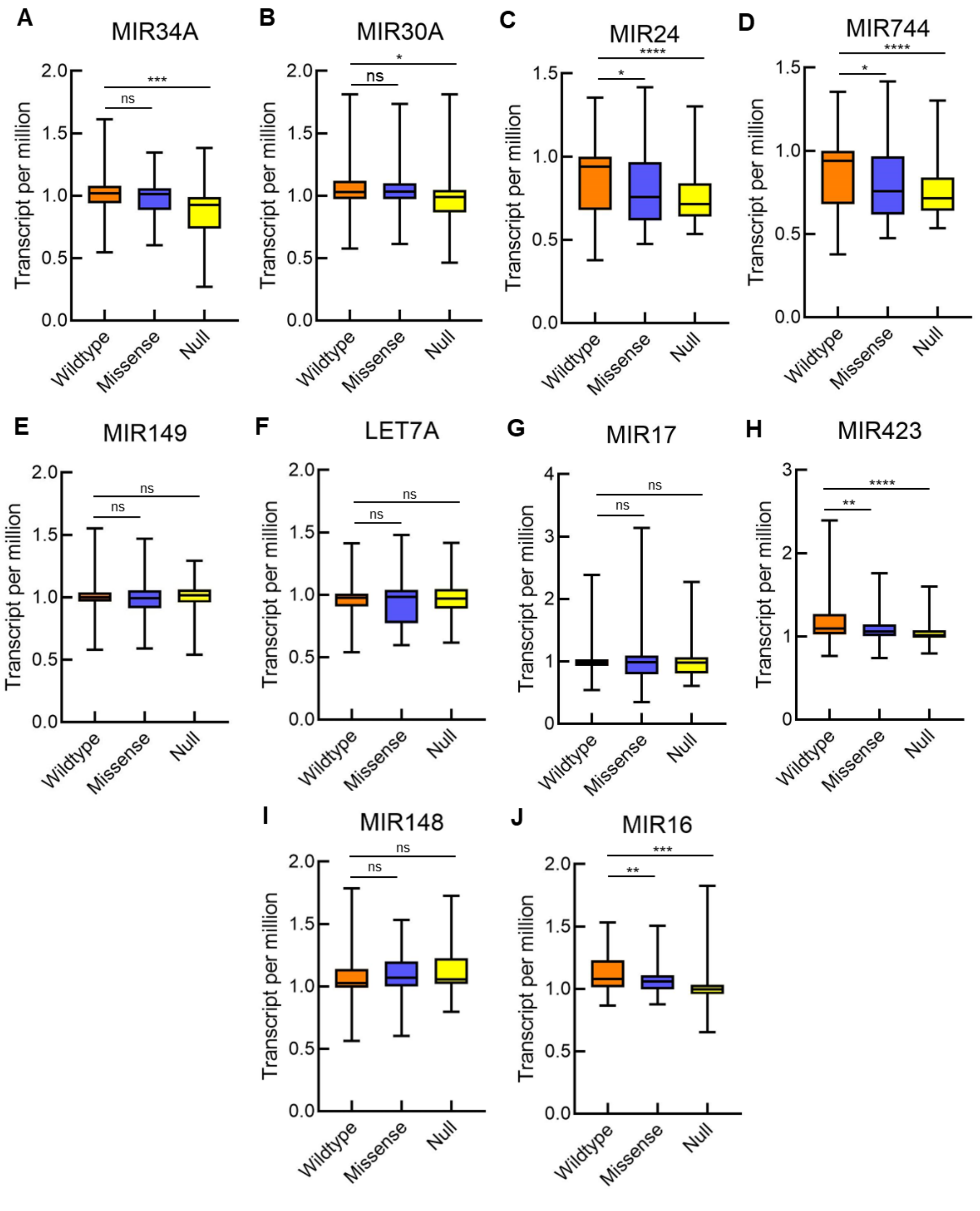

Figure S9: LIHC samples from TCGA were divided into WT p53, p53-null (frameshift and nonsense TP53 mutations), and p53 missense mutated tumors. The transcriptional levels of mir-34a (A), mir-30a (B), mir-24 (C), mir-744 (D), mir-149 (E), let-7a (F), mir-17 (G), mir-423 (H), mir-148 (I), and mir-16 (J) in wildtype TP53 (Orange), p53 missense mutated (Blue), and p53 null (Yellow) LIHC tumors.

Table S1: Primer sequences for real time-PCR reactions used in the article.

**Primers**	**Sequence**
hTRIM3-F	GCGACCTGGAGACCATTTGT
hTRIM3-R	GCTACTGCCGATGTGTTCCTG
hTRIM6-F	CTTTCCCACTACTCTTTGTC
hTRIM6-R	TAAGCCTCAGGGTACTTATC
hTRIM11-F	CACCTAAGCTGCACAGTTCC
hTRIM11-R	GGCTGCCTCCTAATTCTTCC
hTRIM24-F	AAAGGACCATCGCATGAAAC
hTRIM24-R	ATGCTGTACTGCTGCCACTG
hTRIM25-F	GTCTCTACCCAGAACAGTTTCC
hTRIM25-R	ATCCAACACAGGCTGATTCC
hTRIM28-F	ATGGTGCAGACAGCACTGG
hTRIM28-R	GCAGTACACGCTCACATTTCC
hTRIM32-F	CCGGGAAGTGCTAGAATGCC
hTRIM32-R	CAGCGGACACCATTGATGCT
hTRIM45-F	AAGATGTCAGAAATCAGGA
hTRIM45-R	GCATCAGAGCGCCACGGTCC
hTRIM59-F	CCTGTGTTTGAGATAGATTTAAGAGC
hTRIM59-R	GCAACAAGGTGAGACCCAGT
