# Supplementary figures and images for: Comprehensive Analysis of the Prognostic Significance of the TRIM Family in the Context of TP53 Mutations in Cancers
Source: Cancers (Basel). 2023 Jul 26;15(15):3792. doi: 10.3390/cancers15153792 (PMC10417774; doi:10.3390/cancers15153792)

Figure 6A

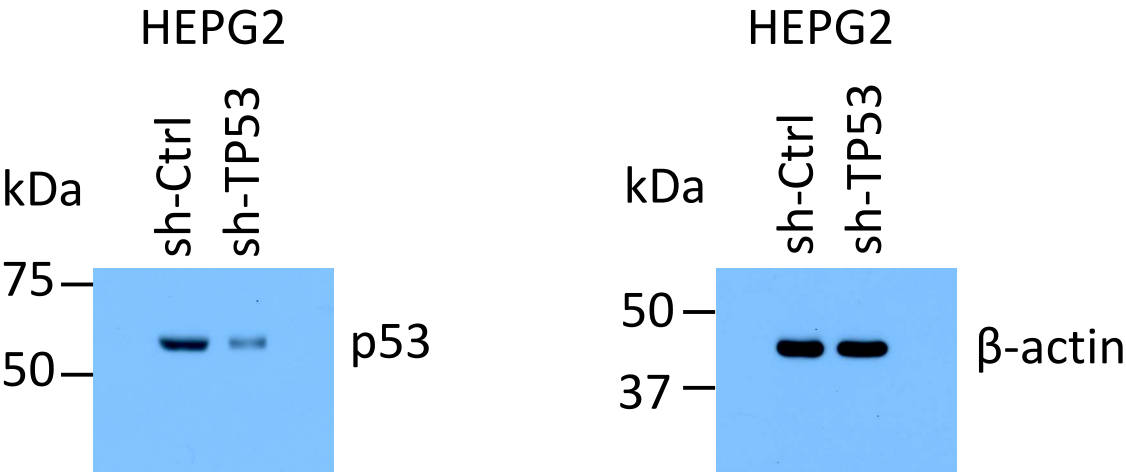

Supplement: Supplementary file 1 [file cancers-15-03792-s001.zip › cancers-2424200-File S1.pdf]
